# Supplementary figures and images for: Modulation of Staphylococcus aureus gene expression during proliferation in platelet concentrates with focus on virulence and platelet functionality
Source: PLoS One. 2024 Jul 25;19(7):e0307920. doi: 10.1371/journal.pone.0307920 (PMC11271859; doi:10.1371/journal.pone.0307920)

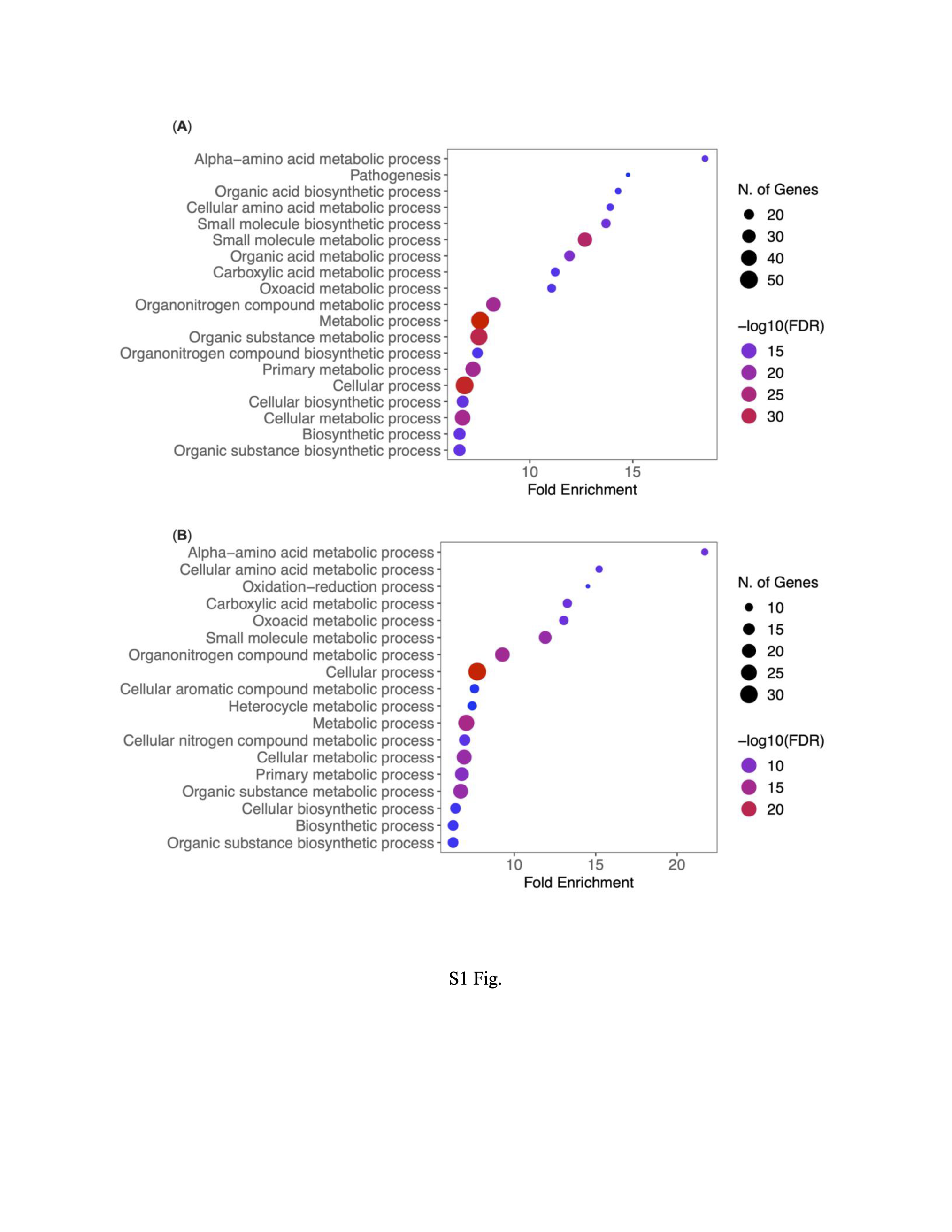

Supplement: S1 Fig — Significantly enriched pathways in S. aureus CBS2016-05 and PS/BAC/317/16/W strains presented as Dot plots with a p. adjust threshold cut-off of 0.05. Enrichment significance is indicated by bubble color, while bubble size corresponds to gene count in the term. (TIF) [file pone.0307920.s001.tif]
