# Supplementary material for: Modulation of Staphylococcus aureus gene expression during proliferation in platelet concentrates with focus on virulence and platelet functionality
Source: PLoS One. 2024 Jul 25;19(7):e0307920. doi: 10.1371/journal.pone.0307920 (PMC11271859; doi:10.1371/journal.pone.0307920)
Supplement: Attachment — Submitted filename: Response to Reviewers.docx [file pone.0307920.s004.docx]

Reviewer 1

The manuscript is presented in an intelligible fashion and written in standard English. A few minor changes are suggested:

- Line 210: It would be interesting to know how platelet counts in the units correlate with flow analysis of activation and apoptosis indicators.

Response: Platelet counts for activation and apoptosis assays were performed with samples containing 10-40 x 10^6 platelets/mL, which are the platelet counts required for optimal performance of these assays.

- It is interesting to know the K levels, PH value and external inspection of the units (presence of clots) of the tested units in correlation with the gene expression as you found for example that for the CBS2016-05 strain, an upregulation of up to 7-fold was observed in the potassium transporter operon (kdpABCDE), with two such operons present in this strain (Line 406)

Response: We thank the Reviewer for this important observation. We did not determine pH or K levels in the tested units and therefore cannot directly correlate the upregulation of the *kdp* operon with clot formation. However, this could be one of the explanations for the aggregates observed in the inoculated units. We added the potential for this correlation in the discussion of the revised manuscript (lines 420-423).

- Please include limitations of this study such as the fact that functionality depends on bacterial density.

Response: This is an important consideration and we have addressed this in the revised manuscript (lines 467-468).

- How might this be the source of delayed detection of PC units, which are typically held in blood banks for 5-7 days. What are the likely causes of missing detection of this bacterium during routine PC screening using automated culture systems?

Response: Platelet concentrates screening is done at approximately 36 hours post blood collection. Bacterial titers are typically very low (<0.01 CFU/ml) in platelet concentrates and if the isolate is a slow grower or a biofilm former, the titer could be under the limit of the detection of culture methods when sampling is done. We have added a sentence to address this question in the revised manuscript (lines 510 and 511).

EDITOR :

1. Please ensure that your manuscript meets PLOS ONE's style requirements [https://journals.plos.org/plosone/s/file?id=wjVg/PLOSOne_formatting_sample_main_body.pdf](https://can01.safelinks.protection.outlook.com/?url=https%3A%2F%2Fjournals.plos.org%2Fplosone%2Fs%2Ffile%3Fid%3DwjVg%2FPLOSOne_formatting_sample_main_body.pdf&data=05%7C02%7Cbyousuf%40uottawa.mail.onmicrosoft.com%7C870ddca489a641e4f62a08dc8bb0db63%7Cd41fdab17e154cfdb5fa7200e54deb6b%7C1%7C0%7C638538839139028087%7CUnknown%7CTWFpbGZsb3d8eyJWIjoiMC4wLjAwMDAiLCJQIjoiV2luMzIiLCJBTiI6Ik1haWwiLCJXVCI6Mn0%3D%7C0%7C%7C%7C&sdata=F9Ytf%2BQR%2Ba0%2Bp3YDHakc6VcbhLb2t2HQXi9GqKeFOl4%3D&reserved=0) and
   [https://journals.plos.org/plosone/s/file?id=ba62/PLOSOne_formatting_sample_title_authors_affiliations.pdf](https://can01.safelinks.protection.outlook.com/?url=https%3A%2F%2Fjournals.plos.org%2Fplosone%2Fs%2Ffile%3Fid%3Dba62%2FPLOSOne_formatting_sample_title_authors_affiliations.pdf&data=05%7C02%7Cbyousuf%40uottawa.mail.onmicrosoft.com%7C870ddca489a641e4f62a08dc8bb0db63%7Cd41fdab17e154cfdb5fa7200e54deb6b%7C1%7C0%7C638538839139042630%7CUnknown%7CTWFpbGZsb3d8eyJWIjoiMC4wLjAwMDAiLCJQIjoiV2luMzIiLCJBTiI6Ik1haWwiLCJXVCI6Mn0%3D%7C0%7C%7C%7C&sdata=oBPc%2Bpdha3b8KPrToHEVnEAEvawwrunQHanFVyulDYQ%3D&reserved=0)

Response: The manuscript complies with PLOS ONE’ style.

2. We suggest you thoroughly copyedit your manuscript for language usage, spelling, and grammar

Response: The manuscript has been reviewed for proper language usage, spelling and grammar.

3. Thank you for stating the following financial disclosure: "The project was funded by Canadian Blood Services (intramural grant awarded to Sandra Ramirez-Arcos) and Health Canada. "
   Please state what role the funders took in the study.  If the funders had no role, please state: ""The funders had no role in study design, data collection and analy sis, decision to publish, or preparation of the manuscript."" 
   If this statement is not correct you must amend it as needed. 
   Please include this amended Role of Funder statement in your cover letter; we will change the online submission form on your behalf.

Response: In cover letter, we have added the statement “The funders had no role in study design, data collection and analysis, decision to publish, or preparation of the manuscript”.

4. We note that you have included the phrase “data not shown” in your manuscript. Unfortunately, this does not meet our data sharing requirements. PLOS does not permit references to inaccessible data.

Response: The phrase “data not shown” has been removed from the manuscript as the statement is supported by our recent paper as a reference.

Please review your reference list to ensure that it is complete and correct.

Response: The reference list and citations in the manuscript have been reviewed.
